# Serum N-terminal Pro-B-type Natriuretic Peptide Predicts Mortality in Cardiac Surgery Patients Receiving Renal Replacement Therapy

**DOI:** 10.3389/fmed.2020.00153

**Published:** 2020-05-08

**Authors:** Ying Su, Jun-yi Hou, Yi-jie Zhang, Guo-guang Ma, Guang-wei Hao, Jing-chao Luo, Zhe Luo, Guo-wei Tu

**Affiliations:** ^1^Department of Critical Care Medicine, Zhongshan Hospital, Fudan University, Shanghai, China; ^2^Department of Critical Care Medicine, Xiamen Branch, Zhongshan Hospital, Fudan University, Xiamen, China

**Keywords:** acute kidney injury, renal replacement therapy, biomarker, N-terminal pro-B-type natriuretic peptide, mortality

## Abstract

**Background:** N-terminal pro-B-type natriuretic peptide (NT-proBNP) is a useful cardiac biomarker that is associated with acute kidney injury (AKI) and mortality after cardiac surgery. However, its prognostic value in cardiac surgical patients receiving renal replacement therapy (RRT) remains unclear.

**Objectives:** Our study aimed to assess the prognostic value of NT-proBNP in patients with established AKI receiving RRT after cardiac surgery.

**Methods:** A total of 163 cardiac surgical patients with AKI requiring RRT were enrolled in this study. Baseline characteristics, hemodynamic variables at RRT initiation, and NT-proBNP level before surgery, at RRT initiation, and on the first day after RRT were collected. The primary outcome was 28-day mortality after RRT initiation.

**Results:** Serum NT-proBNP levels in non-survivors was markedly higher than survivors before surgery (median: 4,096 [IQR, 962.0–9583.8] vs. 1,339 [IQR, 446–5,173] pg/mL; *P* < 0.01), at RRT initiation (median: 10,366 [IQR, 5,668–20,646] vs. 3,779 [IQR, 1,799–11,256] pg/mL; *P* < 0.001), and on the first day after RRT (median: 9,055.0 [IQR, 4,392–24,348] vs. 5,255 [IQR, 2,134–9,175] pg/mL; *P* < 0.001). The area under the receiver operating characteristic curve of NT-proBNP before surgery, at RRT initiation, and on the first day after RRT for predicting 28-day mortality was 0.64 (95% CI, 0.55–0.73), 0.71 (95% CI, 0.63–0.79), and 0.68 (95% CI, 0.60–0.76), respectively. Consistently, Cox regression revealed that NT-proBNP levels before surgery (HR: 1.27, 95% CI, 1.06–1.52), at RRT initiation (HR: 1.11, 95% CI, 1.06–1.17), and on the first day after RRT (HR: 1.17, 95% CI, 1.11–1.23) were independently associated with 28-day mortality.

**Conclusions:** Serum NT-proBNP was an independent predictor of 28-day mortality in cardiac surgical patients with AKI requiring RRT. The prognostic role of NT-proBNP needs to be confirmed in the future.

## Introduction

Acute kidney injury (AKI) is a frequent but serious complication for patients undergoing cardiac surgery, with an increased risk of hospital mortality and prolonged length of hospital stay (1). Patients who develop severe AKI requiring renal replacement therapy (RRT) represent nearly 2–6% of patients after cardiac surgery, and RRT dependency results in high mortality (2–6). However, few biomarkers and effective scoring systems have been validated as prognostic factors in this high risk population (7, 8).

Serum N-terminal pro-B-type natriuretic peptide (NT-proBNP), as an inactive polypeptide of the pre-prohormone brain natriuretic peptide (BNP), is synthesized and released by cardiomyocytes in response to pressure and volume overload (9–11). Increasing evidence has shown that the NT-proBNP level is associated with AKI after cardiac surgery (12–15), medical (non-surgical) patients in cardiac intensive care units (16), or unselected critically ill patients (17). Several studies have also validated NT-proBNP as a predictor of mortality in cardiac surgery patients (12, 14, 18, 19). However, little is known about the prognostic value of NT-proBNP in cardiac surgery patients with established AKI. Therefore, the purpose of this study was to investigate the prognostic value of NT-proBNP in cardiac surgery patients with established AKI requiring RRT.

## Materials and Methods

### Study Population

From January 2018 to October 2019, consecutive AKI patients after cardiac surgery who required RRT in the cardiac surgical intensive care unit (ICU) of Zhongshan Hospital, Fudan University, Shanghai, China, were prospectively enrolled.

AKI was diagnosed according to the Kidney Disease Improving Global Outcomes (KDIGO) classification (20). Patients were excluded if they met the following criteria: age < 18 years, ICU length of stay < 48 h, pre-admission chronic RRT or previous history of end-stage renal disease [defined by an estimated glomerular filtration rate (eGFR) < 15 mL/min/1.73 m^2^], and receiving RRT before cardiac surgery.

### RRT Indications and Management

Indications for RRT included metabolic acidosis (pH < 7.2), hyperkalemia > 6.0 mmol/L, evidence of fluid overload refractory to diuretics, urine output < 0.5 mL/kg/h for more than 6 h under optimized conditions (preload optimization, titration of vasopressors, and use of diuretics), and severe azotemia (serum creatinine level > 4 mg/dL and/or >3-fold increase in serum creatinine level compared with baseline). RRT was initiated within 6 h of meeting the above criteria. The method of RRT (continuous or intermittent technique, duration, and interval between sessions) depended on the clinical state of individual patients, usually the continuous technique in unsteady hemodynamic phase and then succeeded by intermittent techniques after stabilization. The modality of RRT included continuous venovenous hemofiltration (CVVH), continuous venovenous hemodiafiltration (CVVHDF), intermittent hemodialysis (IHD), and intermittent sustained low efficiency dialysis (SLED), which were used based on the discretion of clinicians to achieve optimal hemodynamic status and metabolic control. Blood flow was usually kept between 180 to 220 mL per minute. The prescribed effluent flow was kept above 25 mL per kilogram per hour. The replacement or dialysate solution used was bicarbonate. Femoral or internal jugular double-lumen dialysis catheter was used for vascular access.

### Data Collection

Baseline demographics, co-morbidities, and pre-operative laboratory data including NT-proBNP, serum creatinine (sCr), and blood urea nitrogen (BUN) were recorded. Information regarding the surgical procedure was obtained. The eGFR was calculated based on the Modification of Diet in Renal Disease (MDRD) equation. Day 0 was defined as the day of RRT initiation, and day 1 was defined as the first day after RRT initiation. RRT indications, hemodynamic variables, and clinical characteristics at RRT initiation which included central venous pressure (CVP), mean artery pressure (MAP), vasoactive agent dosages, and Acute Physiology and Chronic Health Evaluation II (APACHE-II) scores were collected.

Serum samples for laboratory assessments were obtained for each patient on day 0 (within 6 h before RRT initiation) and day 1 (within 18 h after RRT initiation). The NT-proBNP levels were measured using the Elecsys Electro-chemo luminescent assay (Cobase 411 analyzer, Roche Diagnostics, Mannheim, Germany) in the clinical chemistry laboratory of the Zhongshan Hospital. The measurement range was 5 to 35,000 pg/mL and the total coefficient of variation was 3.9–4.4% according to multicenter measurements of the automated Roche NT-proBNP assay (21).

### Outcome

The primary outcome of this study was 28-day mortality from the day of RRT initiation. Patients who survived to day 28 were censored at day 28. Secondary outcomes were duration of invasive mechanical ventilation, length of stay in the ICU and hospital, ICU mortality, hospital mortality, and RRT dependency at day 28 in survivors.

### Statistical Analysis

The normality of distribution of continuous variables was evaluated using the Kolmogorov-Smirnov test. Continuous variables are shown as the mean ± standard deviation (SD) or median interquartile range [IQR], as appropriate. Categorical variables were presented as numbers and percentages. For skewed data, ln transformation of NT-proBNP, troponin T, and APACHE II score was performed (presented as ln-NT-proBNP, ln-troponin T, and ln-APACHE II score, respectively). Baseline characteristics were compared using the Student's *t-*test or Mann-Whitney *U*-test for continuous variables and the chi-square test or Fisher's exact test for categorical variables. We used receiver operating characteristic (ROC) curves to examine the performance of variables to predict 28-day mortality. The area under the curve (AUC) was derived from ROC curves. Survival curves were plotted and compared across different NT-proBNP levels. Cox proportional hazards regressions were performed to evaluate prognostic values of NT-proBNP. Variables with a *P* < 0.1 in univariate analyses were introduced into multivariable models (stepwise variable-selection method). Analyses were performed using the SPSS software package, version 13.0 (SPSS, Inc., Chicago, IL, USA). Two-sided *p* < 0.05 was defined as statistically significant.

## Results

**Figure 1:**
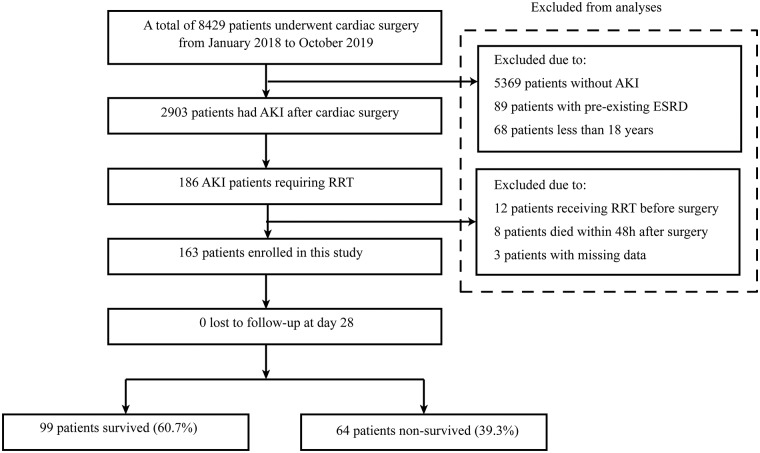
Flow chart of this study. AKI, acute kidney injury; RRT, renal replacement therapy; ESRD, end-stage renal disease.

From January 2018 to October 2019, a total of 8,429 patients undergoing cardiac surgery were screened for inclusion. Of these, 2,903 patients had AKI after cardiac surgery and 186 patients required RRT. Of those, 23 patients were excluded, including 12 patients receiving RRT before surgery, eight patients who died within 48 h after surgery, and three patients with missing data. Finally, 163 patients that received RRT were included for analysis (Figure 1).

### Patient characteristics

The baseline characteristics grouped by 28-day mortality are presented in Table 1. There were no significant differences in age, sex, hypertension, and diabetes mellitus. Non-survivors had higher pre-operative troponin *T* (median: 0.03 [IQR, 0.02–0.14] vs. 0.02 [IQR, 0.01–0.05] ng/mL; *P* = 0.03), NT-proBNP (median: 4,096 [IQR, 962.0–9,583.8] vs. 1,339 [IQR, 446–5,173] pg/mL; *P* < 0.01), sCr levels (mean: 173.4, SD ± 92.1 vs. 141.3 SD ± 78.1 μmol/L; *P* = 0.03) and lower BMI (mean: 22.7, SD ± 4.0 vs. 24.1, SD ± 4.4 kg/m^2^; *P* = 0.04) compared with survivors. The type of surgery was comparable between the two groups. The cardiopulmonary bypass (CPB) time and aortic clamp time were higher in non-survivors compared to survivors (all *P* < 0.05).

**Table 1:** Clinical characteristics of patients grouped by 28-day mortality.

	**All patients (*n* = 163)**	**Survivors (*n* = 99)**	**Non-survivors (*n* = 64)**	***P*-value**
Age (years)	56.5 ± 14.7	56.7 ± 14.4	56.3 ± 15.3	0.89
Sex (male), *n* (%)	110 (67.5)	68 (68.7)	42 (65.6)	0.73
BMI (kg/m^2^)	23.5 ± 4.3	24.1 ± 4.4	22.7 ± 4.0	0.04
**Comorbidities**				
Hypertension, *n* (%)	103 (63.2)	60 (60.6)	43 (67.2)	0.41
Diabetes mellitus, *n* (%)	25 (15.3)	16 (16.2)	9 (14.1)	0.83
CAD, *n* (%)	27 (16.6)	12 (12.1)	15 (23.4)	0.08
Prior cardiac surgery, *n* (%)	37 (22.7)	25 (25.3)	12 (18.8)	0.44
**Pre-operative laboratory data**				
BUN_pre−op_ (mmol/L)	12.9 ± 10.4	11.7 ± 7.4	14.7 ± 13.6	0.15
cTnT_pre−op_ (ng/mL)	0.03 [0.01,0.06]	0.02 [0.01,0.05]	0.03 [0.02,0.14]	0.03
NT-proBNP_pre−op_ (pg/mL)	1924 [523,7277]	1339 [446,5173]	4096 [962.0,9583.8]	<0.01
sCr_pre−op_ (μmol/L)	154.41 ± 85.32	141.3 ± 78.1	173.4 ± 92.1	0.03
Procalcitonin_pre−op_ (ng/mL)	0.23 [0.05,0.68]	0.07 [0.05,0.44]	0.25 [0.10,1.00]	0.10
eGFR_pre−op_ (MDRD) (mL/min/1.73 m^2^)	54.00 ± 32.13	58.1 ± 33.0	48.0 ± 30.1	0.06
**Type of surgery**, ***n*** **(%)**				
CABG only	14 (8.6)	8 (8.1)	16 (25.0)	0.78
Valve only	58 (35.6)	37 (37.4)	21 (32.8)	0.62
CABG and valve	9 (5.5)	4 (4.0)	5 (7.8)	0.32
Aortic surgery	41 (25.2)	27 (27.3)	14 (21.9)	0.47
Valve and large vessels	20 (12.3)	13 (13.1)	7 (10.9)	0.81
Other cardiac surgery	21 (12.9)	10 (1.1)	11 (17.2)	0.23
**Intraoperative parameters**				
CPB time (min)	193 [152,238]	182 [148,227]	206 [161,252]	0.03
Aortic clamp time (min)	75 [60,93]	73 [58,90]	82 [65,94]	0.04

*Continuous variables are shown as the mean (±SD) or median [interquartile range, IQR], as appropriate. Categorical variables are shown as a number (%). BMI, body mass index; BUN, blood urea nitrogen; CAD, coronary artery disease; CABG, coronary artery bypass grafting; CPB, cardiopulmonary bypass; sCr, serum creatinine; eGFR, estimated glomerular filtration rate using the abbreviated Modification of Diet in Renal Disease (MDRD) equation; cTnT, troponin T; NT-proBNP, N-terminal pro-B-type natriuretic peptide; pre-op, pre-operative*.

The clinical characteristics at RRT initiation are shown in Table 2. The indications for RRT included severe azotemia (25.8%), oliguria (97.5%), metabolic acidosis (6.1%), and electrolyte disorders (3.1%). There were no differences in indications of RRT, urine output before RRT initiation, and hemodynamic variables including MAP, CVP, and the dosage of vasoactive drugs between two groups. The APACHE II score (mean: 18, SD ± 8 vs. 15, SD ± 6; *P* < 0.01), NT-proBNP (median: 10,366 [IQR, 5,668–20,646] vs. 3,779 [IQR, 1,799–11,256] pg/mL; *P* < 0.001) and serum lactate level (mean: 5.88, SD ± 5.19 vs. 3.76, SD ± 3.63 mmol/L; *P* < 0.01) at RRT initiation were higher in non-survivors, while the eGFR (mean: 20.9, SD ± 10.3 vs. 25.2, SD ± 15.7 mL/min/1.73 m^2^; *P* = 0.04) and serum bicarbonate level (mean: 23.2, SD ± 4.1 vs. 25.7, SD ± 4.2 mmol/L; *P* < 0.001) were lower. On day 1 after RRT initiation, NT-proBNP (median: 9,055.0 [IQR, 4,392–24,348] vs. 5,255 [IQR, 2,134–9,175] pg/mL; *P* < 0.001) and serum lactate level (mean: 4.74, SD ± 4.68 vs. 1.90, SD ± 1.30 mmol/L; *P* < 0.001) were still higher in non-survivors, while serum bicarbonate level (mean: 23.0, SD ± 4.2 vs. 25.6, SD ± 3.7 mmol/L; *P* < 0.001) was lower.

**Table 2:** Patient characteristics at RRT initiation grouped by 28-day mortality.

	**All patients (*n* = 163)**	**Survivors (*n* = 99)**	**Non-survivors (*n* = 64)**	***P*-value**
LOS before RRT initiation (day)	1 [1, 3]	1 [1, 2]	1.5 [1, 7.8]	0.02
**Indication for RRT**				
Severe azotemia, *n* (%)	42 (25.8)	21 (21.2)	21 (32.8)	0.10
Oliguria, *n* (%)	159 (97.5)	96 (97.0)	63 (98.4)	1.00
Metabolic acidosis, *n* (%)	10 (6.1)	4 (4.0)	6 (9.4)	0.19
Electrolyte disorders, *n* (%)	5 (3.1)	2 (2.0)	3 (4.7)	0.38
Urine output before RRT initiation (mL/kg/h)	0.45 ± 0.24	0.45 ± 0.25	0.45 ± 0.23	0.96
Patients on IABP, *n* (%)	8 (4.9)	4 (4.0)	4 (6.3)	0.71
Patients on ECMO, *n* (%)	26 (15.9)	9 (9.1)	17 (26.6)	<0.01
Heart rate (bpm)	106.8 ± 18.5	105.6 ± 19.1	108.6 ± 17.4	0.30
Mean arterial pressure (mmHg)	75.0 ± 15.1	75.2 ± 14.9	74.8 ± 15.6	0.87
CVP (mmHg)	17 ± 4	17 ± 4	17 ± 3	0.66
Norepinephrine dose (μg/kg/min)	0.19 [0.11, 0.20]	0.19 [0.10, 0.28]	0.20 [0.12, 0.26]	0.89
Epinephrine dose (μg/kg/min)	0.15 [0.09, 0.27]	0.15 [0.06, 0.27]	0.18 [0.10, 0.27]	0.11
APACHE II score	16 ± 7	15 ± 6	18 ± 8	<0.01
RRT dose (mL/kg/h)	38.34 ± 11.14	37.50 ± 11.21	39.63 ± 11.00	0.23
**Laboratory data at RRT initiation**				
BUN_day0_ (mmol/L)	20.6 ± 13.2	19.0 ± 10.4	23.5 ± 16.7	0.08
cTnT_day0_ (ng/mL)	1.20 [0.36, 2.54]	1.21 [0.54, 2.40]	0.94 [0.21, 4.38]	0.47
NT-proBNP_day0_ (pg/mL)	5,876 [2,221, 12,816]	3,779 [1,799, 11,256]	10,366 [5,668, 20,646]	<0.001
sCr_day0_ (μmol/L)	286.51 ± 12.02	276.5 ± 124.4	302.0 ± 112.2	0.19
Procalcitonin_day0_ (ng/mL)	4.16 [1.53, 13.56]	4.15 [1.49, 11.79]	4.20 [1.94, 17.28]	0.50
eGFR_day0_ (MDRD) (mL/min/1.73 m^2^)	23.47 ± 13.97	25.2 ± 15.7	20.9 ± 10.3	0.04
Bicarbonate_day0_ (mmol/L)	24.7 ± 4.4	25.7 ± 4.2	23.2 ± 4.1	<0.001
Lactate_day0_ (mmol/L)	4.60 ± 4.42	3.76 ± 3.63	5.88 ± 5.19	<0.01
**Laboratory data on the first day after RRT initiation**				
BUN_day1_ (mmol/L)	15.95 ± 7.21	15.5 ± 6.3	16.6 ± 8.45	0.41
cTnT_day1_ (ng/mL)	1.20 [0.60, 3.18]	1.14 [0.58, 2.86]	1.27 [0.61, 3.31]	0.51
NT-proBNP_day1_ (pg/mL)	6,494 [2,684, 12,817]	5,255 [2,134, 9,175]	9055.0 [4,392, 24,348]	<0.001
sCr_day1_ (μmol/L)	250.27 ± 95.16	247.0 ± 88.0	255.3 ± 105.8	0.60
Procalcitonin_day1_ (ng/mL)	6.25[2.20, 17.76]	5.75[1.97, 17.04]	7.32[3.45, 19.67]	0.35
eGFR_day1_ (MDRD) (mL/min/1.73 m^2^)	26.8 ± 16.2	27.3 ± 18.0	26.1 ± 13.1	0.65
Bicarbonate_day1_ (mmol/L)	24.6 ± 4.1	25.6 ± 3.7	23.0 ± 4.2	<0.001
Lactate_day1_ (mmol/L)	3.09 ± 3.47	1.90 ± 1.30	4.74 ± 4.68	<0.001

*Results are presented as the mean (± SD) or median [IQR], as appropriate. LOS, length of stay; NT-proBNP, N-terminal pro-B-type natriuretic peptide; cTnT, troponin T; BUN, blood urea nitrogen; sCr, serum creatinine; eGFR, estimated glomerular filtration rate using the abbreviated Modification of Diet in Renal Disease (MDRD) equation; RRT, renal replacement therapy; CVP, central venous pressure; ECMO, extracorporeal membrane oxygenation; IABP, intra-aortic balloon pump. Day 0 was defined as the day at RRT initiation, while day 1 was defined as the first day after RRT initiation*.

### Outcome

The 28-day mortality of our cohort was 39.3% (64/163; Table 3). Concerning the clinical course, 43.6% patients (71/163) received tracheostomy during the ICU stay. The mean duration of invasive mechanical ventilation was 11, SD ± 11 days. The rate of dependence on RRT among survivors at 28 days was 12.1% (12/99). The length of ICU and hospital stay were longer in non-survivors compared with survivors (mean: 21.0, SD ± 16.0 vs.14, SD ± 10 days and 39, SD ± 26 vs. 21, SD ± 13 days, all *P* < 0.01; Table 3).

**Table 3:** Clinical outcome grouped by 28-day mortality.

	**All patients (*n* = 163)**	**Survivors (*n* = 99)**	**Non-survivors (*n* = 64)**	***P*-value**
Patients on tracheostomy, *n* (%)	71 (43.6)	40 (40.4)	31 (48.4)	0.34
Duration of IMV (day)	11 ± 11	11 ± 12	11 ± 7	0.78
Dependence on RRT among survivors at day 28, *n* (%)	/	12 (12.1%)	/	/
ICU mortality, *n* (%)	73 (44.8)	9 (9.1)	64 (100)	<0.001
Hospital mortality, *n* (%)	73 (44.8)	9 (9.1)	64 (100)	<0.001
Length of ICU stay (day)	18 ± 14	21 ± 16	14 ± 10	0.001
Length of hospital stay (day)	32 ± 23	39 ± 26	21 ± 13	<0.001

*Results are presented as the mean (±SD). RRT, renal replacement therapy; IMV, invasive mechanical ventilation*.

### Value of Variables to Predict 28-Day Mortality

**Figure 2:**
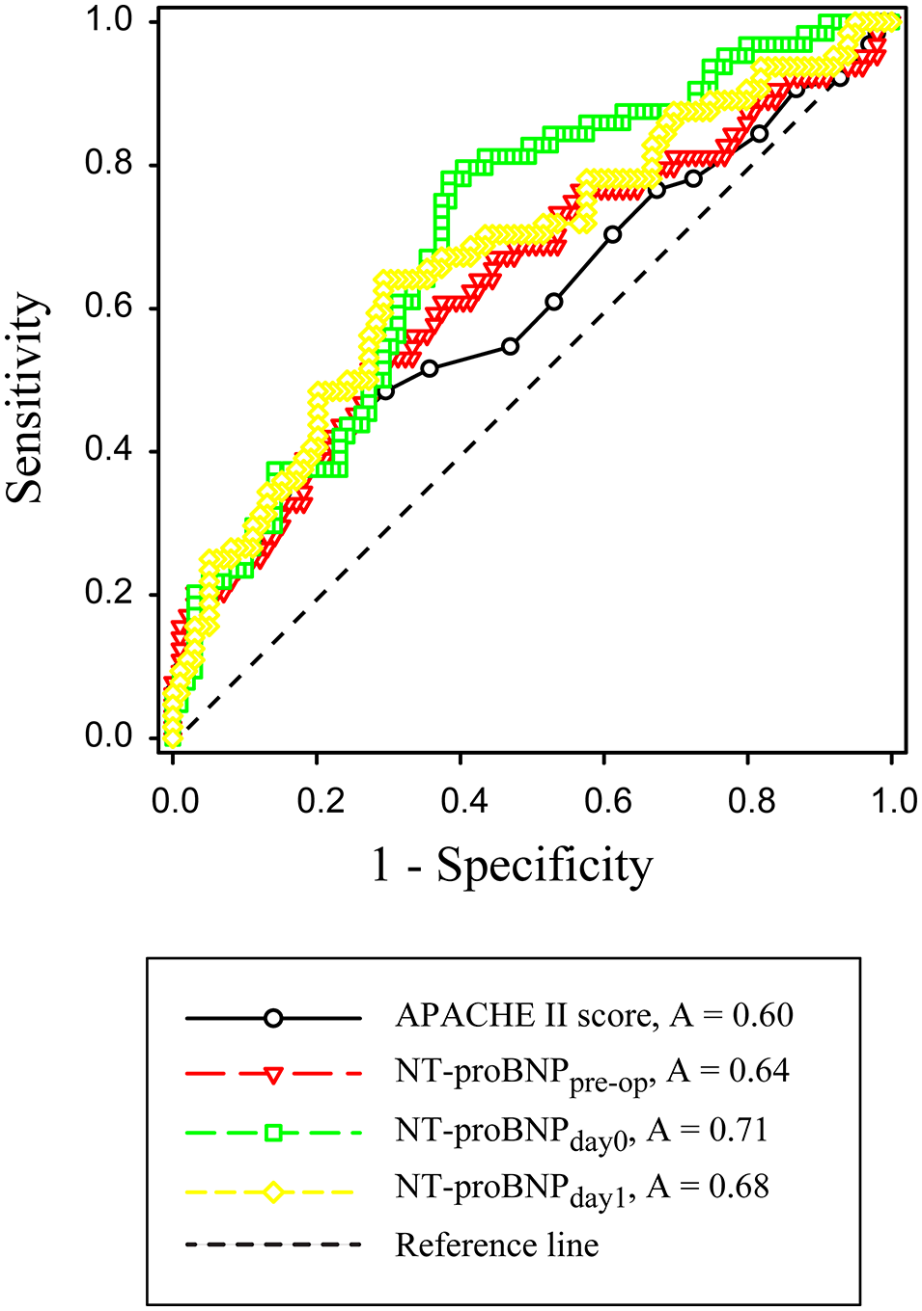
Receiver operating characteristic curves for APACHE II score, NT-proBNP_pre−op_, NT-proBNP_day0_ and NT-proBNP_day1_. APACHE-II, Acute Physiology and Chronic Health Evaluation II; NT-proBNP_pre−op_, preoperative NT-proBNP level; NT-proBNP_day0_, NT-proBNP level at RRT initiation; NT-proBNP_day1_, NT-proBNP level on the first day after RRT initiation.

ROC curves were constructed to evaluate the performance of variables to predict 28-day mortality (Figure 2). The AUC, optimal cutoff value, sensitivity, and specificity of each variable are shown in Table 4. The APACHE II score (AUC 0.60, SD ± 0.05), NT-proBNP_pre−op_ (AUC 0.64, SD ± 0.05), NT-proBNP_day0_ (AUC 0.71, SD ± 0.04), and NT-proBNP_day1_ (AUC 0.68, SD ± 0.04) had a modest power for prediction of 28-day mortality (all *P* < 0.05). There were no statistically significant differences in the AUC among above indicators (all *P* > 0.05). A NT-proBNP_pre−op_ cutoff value of ≥3,632.5 pg/mL had a sensitivity of 53.1% and a specificity of 71.4%. A NT-proBNP_day0_ threshold of ≥5,539 pg/mL had a sensitivity of 78.1% and a specificity of 62.2%. A NT-proBNP_day1_ threshold of ≥7,841 pg/mL had a sensitivity of 64.1% and a specificity of 71.4%.

**Table 4:** Performance of variables in predicting 28-day mortality.

	**AU ROC**	**95% CI**	**P**	**Cut-off**	**Sensitivity (%)**	**Specificity (%)**
APACHE II score	0.60 ± 0.05	0.51–0.69	0.032	≥19.5	37.5	81.6
NT-proBNP_pre−op_	0.64 ± 0.05	0.55–0.73	0.003	≥3632.5	53.1	71.4
NT-proBNP_day0_	0.71 ± 0.04	0.63–0.79	0.000	≥5,539	78.1	62.2
NT-proBNP_day1_	0.68 ± 0.04	0.60–0.76	0.000	≥7,841	64.1	71.4

*Results are presented as the mean (±SD). AUROC, area under the receiver operating characteristic curve; CI, confidence interval; Acute Physiology and Chronic Health Evaluation II, APACHE-II; pre-op, preoperative; cTnT, troponin T; NT-proBNP, N-terminal pro-B-type natriuretic peptide. Day 0 was defined as the day at RRT initiation, while day 1 was defined as the first day after RRT initiation*.

### Variables for Prediction of 28-day Mortality

**Figure 3:**
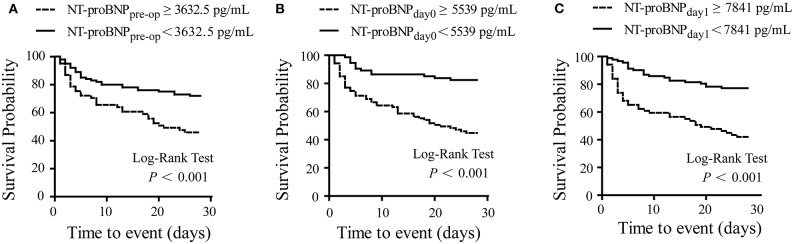
Kaplan-Meier curve for survival. Survival probability in patients with low and high NT-proBNP levels. A significant difference was observed with higher risk of survival events in patients with NT-proBNP_pre−op_ ≥ 3,632.5 pg/mL **(A)**, NT-proBNP_day0_ ≥ 5,539 pg/mL **(B)**, and NT-proBNP_day1_ ≥7,841 pg/mL **(C)**. Log-rank *P*-value shown on graphs. NT-proBNP_pre−op_, preoperative NT-proBNP level; NT-proBNP_day0_, NT-proBNP level at RRT initiation; NT-proBNP_day1_, NT-proBNP level on the first day after RRT initiation.

According to the ROC curves, the cut-off value of NT-proBNP_pre−op_, NT-proBNP_day0_, and NT-proBNP_day1_ levels were set at 3,632.5, 5,539, and 7,841 pg/mL, respectively (Table 4). Patients with higher NT-proBNP_pre−op_ levels (≥3,632.5 pg/mL), NT-proBNP_day0_ (≥5,539 pg/mL), and NT-proBNP_day1_ (≥7,841 pg/mL) levels were at a higher risk of mortality (all *P* < 0.001, log-rank test). Survival curves for 28-day mortality are shown in Figure 3.

The predictive variables of 28-day mortality were evaluated using Cox proportional hazards regression. In univariable analysis, ln-APACHE II score, ln-NT -proBNP_pre−op_, ln-NT -proBNP_day0_, ln-NT -proBNP_day1_, lactate _day0_, lactate _day1_, ${\text{HCO}}_{3}^{-}\text{day}0,$ and ${\text{HCO}}_{3}^{-}\text{day}1$ were significantly associated with 28-day mortality (Table 5). To account for the possible influence of NT-proBNP levels on 28-day mortality, multivariable models were constructed (Table 6). In multivariable analysis, ln NT -proBNP_pre−op_, ln NT-proBNP_day0_, and ln NT-proBNP_day1_ were independently associated with 28-day mortality (all *P* < 0.05).

**Table 5:** Predictors of 28-day mortality by univariate Cox regression analysis.

**Variables**	**Standard β**	**Hazard ratio**	**95% CI**	***P*-value**
BMI	−0.07	0.93	0.87–0.99	0.03
CAD	0.66	1.93	1.08–3.44	0.03
Ln-APACHE II score	0.75	2.11	1.17–3.82	0.01
eGFR_pre−op_	−40.007	0.99	0.99–1.00	0.13
eGFR_day0_	−0.016	0.98	0.96–1.01	0.13
eGFR_day1_	−0.003	0.99	0.98–1.01	0.71
Ln-NT-proBNP_pre−op_	0.23	1.26	1.07–1.48	<0.01
Ln-cTnT_pre−op_	0.14	1.15	0.99–1.35	0.08
Ln-NT-proBNP_day0_	0.54	1.71	1.34–2.18	<0.001
Bicarbonate_day0_	−0.11	0.89	0.84–0.95	<0.001
Lactate_day0_	0.08	1.08	1.03–1.14	0.001
Ln-NT-proBNP_day1_	0.47	1.60	1.25–2.05	<0.001
Bicarbonate_day1_	−0.18	0.83	0.78–0.89	<0.001
Lactate_day1_	0.17	1.18	1.12–1.25	<0.001

*CI, Confidence; BMI, body mass index; CAD, coronary artery disease; APACHE-II, Acute Physiology and Chronic Health Evaluation II; eGFR, estimated glomerular filtration rate using the abbreviated Modification of Diet in Renal Disease (MDRD) equation; NT-proBNP, N-terminal pro-B-type natriuretic peptide; cTnT, troponin T; pre-op, pre-operative. Ln-variable is the logarithm of the variable. Day 0 was defined as the day at RRT initiation, while day 1 was defined as the first day after RRT initiation*.

**Table 6:** Independent predictors of 28-day mortality by multivariate Cox regression analysis.

	**Hazard ratio (95 % CI)**	***P*-value**
**Model 1**		
CAD	1.91 (1.03–3.52)	0.04
Ln-APACHE II score	2.01 (1.09–3.71)	0.03
Ln-NT-proBNP_pre−op_	1.27 (1.06–1.52)	0.01
**Model 2**		
CAD	2.08 (1.14–3.80)	0.02
Ln-NT-proBNP _day0_	1.90 (1.47–2.47)	<0.001
Lactate_day0_	1.11 (1.06–1.17)	<0.001
**Model 3**		
Ln-NT-proBNP_day1_	1.52 (1.18–1.97)	0.001
Lactate_day1_	1.17 (1.11–1.23)	<0.001

*Model 1 adjusted for BMI, CAD, ln-APACHE II score, ln-NT-proBNP_pre−op_, GFR_pre−op_ and ln-cTnT_pre−op_*.

*Model 2 adjusted for BMI, CAD, ln-APACHE II score, ln-NT-proBNP_day0_, eGFR_day0_, lactate_day0_, bicarbonate_day0_*.

*Model 2 adjusted for BMI, CAD, ln-APACHE II score, ln-NT-proBNP_day1_, eGFR_day1_, lactate_day1_, bicarbonate_day1_*.

*CI, Confidence; BMI, body mass index; CAD, coronary artery disease; APACHE-II, Acute Physiology and Chronic Health Evaluation II; eGFR, estimated glomerular filtration rate using the abbreviated Modification of Diet in Renal Disease (MDRD) equation; NT-proBNP, N-terminal pro-B-type natriuretic peptide; cTnT, troponin T; pre-op, pre-operative. Ln-variable is the logarithm of the variable. Day 0 was defined as the day at RRT initiation, while day 1 was defined as the first day after RRT initiation*.

## Discussion

To our knowledge, this is the first study investigating the prognostic value of NT-proBNP in cardiac surgery patients with established AKI requiring RRT. Serum NT-proBNP levels in non-survivors was markedly higher than survivors before surgery, at RRT initiation and on the first day after RRT initiation. Consistently, ROC curves revealed that NT-pro-BNP levels (pre-op, day 0 or day 1) had a modest power for predicting 28-day mortality. Cox proportional hazards regression analyses revealed that NT-proBNP levels before surgery, at RRT initiation, and on the first day after RRT initiation were independently associated with 28-day mortality.

Recent studies have reported several biomarkers to predict mortality at RRT initiation in critically ill patients. Serum neutrophil gelatinase associated lipocalin (NGAL) at initiation of RRT was identified as a prognostic biomarker in unselected critically ill patients (22). Plasma c-terminal FGF-23 (cFGF-23) at inception of RRT was correlated with higher 90-day overall mortality and predicted worse kidney recovery in survivors in critically ill patients with AKI (23). NT-proBNP and procalcitonin (PCT) have also been identified as prognostic markers in septic AKI patients requiring RRT (24). However, there are no definitive biomarkers for outcome prediction in AKI patients requiring RRT after cardiac surgery.

Natriuretic peptides (NPs), specifically NT-proBNP and B-type natriuretic peptide (BNP), are released by cardiomyocytes in response to stress and pressure overload. NT-proBNP is an inactive N-terminal fragment produced from the cleavage of proBNP (9–11). Elevated NT-proBNP levels are usually associated with cardiac dysfunction or heart failure after cardiac surgery and portend a poor outcome (14, 25–27). Decreases in NT-proBNP during follow-up were associated with reduced morbidity and mortality in patients with heart failure (28–30). In addition, recent evidence showed that NT-proBNP also represents a useful prognostic biomarker in septic patients (31–33), non-cardiac surgery patients (34), patients without heart failure (35), and an unselected cohort of critically ill patients (36, 37).

In the present study, NT-pro-BNP levels (pre-op, day 0 or day 1) had a modest power for prediction of 28-day mortality. Although the AUC of NT-proBNP_day0_ was larger than NT-proBNP_pre−op_ or NT-proBNP_day1_, the differences were not statistically significant. Multivariable COX regression analysis also confirmed that serum NT-proBNP levels (pre-op, day 0 or day 1) were independent predictors of 28-day mortality. Several mechanisms may explain the relationship between NT-proBNP and mortality in cardiac surgery patients requiring RRT. First, elevated NT-proBNP are most likely released from stressed myocardium, suggesting that patients with higher NT-proBNP levels may have more severe cardiac dysfunction (27). In this study, the patients receiving RRT tended to be hemodynamically unstable with vasoactive drugs maintenance and high CVP level at RRT initiation, which indicated a reduced cardiac function. Second, all patients in this study had impaired renal function and low GFR at the initiation of RRT. Elevated NT-proBNP levels in this cohort may be partially due to decreased clearance by the kidneys or decreased renal responsiveness to BNP (9). It has been demonstrated that NT-proBNP is associated significantly with progression to end-stage renal disease in patients with chronic kidney disease (38, 39). Even in critically ill patients without underlying cardiac disease, NT-proBNP was independently associated with the maximum stage of AKI and need for RRT (17), which was correlated with high mortality. Third, inflammatory status might also account for increased levels of BNPs, apart from cardiac and renal dysfunction (40). In this study, non-survivors had longer CPB time and aortic clamp time than survivors, which led to a more severe systemic inflammatory reaction and consequently increased the risk of mortality (41, 42).

Several limitations in this study should be addressed. First, the conclusion may be restricted by the small sample size. Second, as the frequency of NT-proBNP measurement is low, the change in NT-proBNP during the clinical course was not analyzed in this study. Finally, as the cohort included patients with severe AKI requiring RRT, we should be cautious to interpret the prognostic capacity in cardiac surgical patients with mild or moderate AKI.

## Conclusion

Serum NT-proBNP was an independent predictor of 28-day mortality in cardiac surgical patients with AKI requiring RRT. Large studies are needed to validate the prognostic role of NT-proBNP.

## Data Availability Statement

All datasets generated for this study are included in the article/supplementary material.

## Ethics Statement

This prospective study was approved by the Ethics Committee of Zhongshan Hospital, Fudan University (No. B2016-147R) and conducted in accordance with the Declaration of Helsinki. Written informed consent was obtained from legal representatives of participants.

## Author Contributions

YS, GT, and ZL contributed to study design. YS, JH, YZ, GM, GH, and JL contributed to participants enrollment. JH and YZ contributed to study management and data collection. YS and GT contributed to manuscript writing. GT and ZL contributed to data analyses and manuscript revision. All authors have read and approved the final manuscript.

## Conflict of Interest

The authors declare that the research was conducted in the absence of any commercial or financial relationships that could be construed as a potential conflict of interest.
